# Greater return to sports after anterior cruciate ligament reconstruction combined with anterolateral ligament reconstruction compared with anterior cruciate ligament reconstruction alone: A systematic review and meta‐analysis

**DOI:** 10.1002/jeo2.70127

**Published:** 2025-01-05

**Authors:** Imelda Lumban‐Gaol, Dananjaya Putramega, Krisna Yuarno Phatama, Dwikora Novembri Utomo, Nicolaas C. Budhiparama

**Affiliations:** ^1^ Nicolaas Institute of Constructive Orthopaedic Research & Education Foundation for Arthroplasty & Sports Medicine at Medistra Hospital Jakarta Indonesia; ^2^ Department of Orthopaedic and Traumatology, Faculty of Medicine Saiful Anwar General Hospital Universitas Brawijaya Malang Indonesia; ^3^ Department of Orthopaedic and Traumatology, Faculty of Medicine Universitas Airlangga Surabaya Indonesia; ^4^ Department of Orthopaedic and Traumatology Dr. Soetomo General Hospital Surabaya Indonesia; ^5^ Department of Orthopaedic and Traumatology, Faculty of Medicine Universitas Gadjah Mada Yogyakarta Indonesia; ^6^ Department of Orthopaedics Leiden University Medical Centre Leiden The Netherlands

**Keywords:** ACL‐RSI, anterior cruciate ligament reconstruction, anterolateral ligament reconstruction, return to competition, return to sports, Tegner score

## Abstract

**Purpose:**

This study aimed to compare the return to sports, return to competition, Tegner score and anterior cruciate ligament‐return to sports injury (ACL‐RSI) scores between patients who underwent ACL reconstruction (ACLR) combined with anterolateral ligament reconstruction (ALLR) and those who underwent ACLR alone.

**Methods:**

Two independent reviewers conducted a literature search in PubMed (MEDLINE), EMBASE, Google Scholar and the Cochrane Library in July 2024, followed by data extraction and quality assessment. This study followed the Preferred Reporting Items for Systematic Reviews and meta‐analysis guidelines. The return to sports rate, return to competition rate, Tegner score and ACL‐RSI score were compared between patients who underwent primary ACLR with ALLR and those who underwent isolated primary or revision ACLR. The methodological quality of the included studies was assessed via the Cochrane risk‐of‐bias tool and methodological items for nonrandomized studies.

**Results:**

In total, 12,139 studies were screened, and 14 (four randomized controlled trials and 10 nonrandomized studies) studies were ultimately evaluated. Compared with isolated ACLR, ACLR combined with ALLR resulted in a higher rate of return to sports and competition. Nevertheless, no significant differences were found in the Tegner score or ACL‐RSI score between the two groups.

**Conclusion:**

Patients who underwent ACLR in combination with ALLR had higher rates of return to sports and competition, but their Tegner activity and ACL‐RSI scores were similar to those of patients who underwent ACLR alone. This finding may assist surgeons in making decisions when treating patients undergoing ACLR, especially athletes.

**Level of Evidence:**

Level III.

**Abbreviations**
ACL: anterior cruciate ligament
ACLR: anterior cruciate ligament reconstruction
ACL‐RSI: anterior cruciate ligament‐return to sports injury
AEAP: anterolateral extra‐articular procedure
ALL: anterolateral ligament
ALLR: anterolateral ligament reconstruction
BTB: bone–patellar tendon–bone
CI: confidence interval
DB: double bundle
ITB: iliotibial band
LET: lateral extra‐articular tenodesis
LFE: lateral femoral epycondyle
NR: not reported
OR: odds ratio
RCTs: randomized controlled trials
SB: single bundle
ST‐G: semitendinosus‐gracilis

## INTRODUCTION

Anterior cruciate ligament reconstruction (ACLR) has evolved and shown satisfactory outcomes in recent years. Although successful ACLR has been reported, only 82% of patients are able to resume sports activities after ACLR, with 63% returning to their preinjury level and 44% returning to competitive sports [3, 4, 25, 26]. Some rotatory instability persists after ACLR [15, 17, 46, 47], but the reason for this rotatory instability is not completely understood. Several studies have shown that ACLR alone cannot fully restore rotatory knee stability, including during walking [16, 17, 31, 37, 46, 47, 50], and can lead to meniscal or cartilage problems [44]. Therefore, several studies have been conducted to better understand the anatomy and biomechanics of the ACL and pivot‐shift test results [51]. The anterolateral complex of the knee plays a pivotal role in controlling rotational instability. The anterolateral complex consists of the superficial iliotibial band and iliopatellar band, deep ITB (Kaplan fibres, retrograde condylar attachment continuous with the capsulo‐osseous layer) and anterolateral ligament (ALL). Therefore, various surgical techniques have been developed to reconstruct this anterolateral complex. These techniques include peripheral plasty and anterolateral extra‐articular procedures (AEAPs) aimed at addressing this specific issue. The most well‐known type of AEAP is lateral extra‐articular tenodesis (LET). However, LET is a nonanatomic procedure that might alter the biomechanics of the knee. One of the main concerns is over‐constraint due to over‐tensioning of the graft [5, 22, 35], which might increase the pressure in the lateral tibiofemoral compartment and cause arthritis of the lateral tibiofemoral compartment [7] and stiffness [38, 48]. The ALL has a potential role in rotational stability [10, 14, 21], which has led to the development of a new procedure to reconstruct the ALL [13, 33]. Therefore, we focused on the outcome of ACLR combined with ALL in terms of return to sport and Tegner scores.

This systematic review aimed to compare the return to sports, return to competition and Tegner scores associated with combined ACL and ALL reconstruction (ALLR) with those associated with ACLR alone.

## MATERIALS AND METHODS

### Search strategy

This study was performed in accordance with the Preferred Reporting Items for Systematic Reviews and Meta‐Analyses guidelines. In July 2024, two independent reviewers searched PubMed (MEDLINE), EMBASE, Google Scholar and the Cochrane Library. We restricted the search to studies published in the English language within the last 10 years. The databases were searched using the following keywords: (anterior cruciate ligament reconstruction or ACLR) and (ALLR), and (return to sports or return to play). To supplement the electronic database search, the reference lists of relevant articles were cross‐checked to identify additional references of interest. After removing duplicates and excluding articles by title, the full texts of the remaining articles were assessed.

### Selection criteria

The inclusion criteria were comparative studies with return to sports, return to competition and Tegner score as the outcomes and skeletally mature patients with ACL rupture who underwent primary ACLR. The exclusion criteria were reports, systematic reviews, case reports, noncomparative studies, patients with >2 surgically treated knee ligaments and isolated extra‐articular procedures. The titles and abstracts of all the articles were reviewed after applying the selection criteria. The full text of the article was assessed if the abstract was unclear on this detail. Two reviewers independently applied the selection criteria. Any differences in opinion on the importance and relevance of any identified article were resolved through discussion until a consensus was reached.

### Data extraction

Two reviewers independently extracted the following demographic data from the articles: study type, number of patients, primary or revision surgery, ACLR technique, ALLR technique, follow‐up duration and fixation angle. The rate of return to sports or play, rate of return to competition, anterior cruciate ligament‐return to sports injury (ACL‐RSI) and Tegner scores were assessed. The rate of return to sports was defined as the ability of the patients to return to their sports activity level prior to the trauma. The patients were categorized as able or unable to return to sports activity levels prior to the trauma.

### Statistical analysis

Data analyses were performed via Review Manager Version 5.3 (Cochrane Collaboration). Odds ratios (ORs) and their corresponding 95% confidence intervals (CIs) were calculated for dichotomous data, including the overall rate of return to sports. Standardized mean differences and 95% CIs were calculated for continuous variables of the Tegner and ACL‐RSI scores. A fixed‐effects or random‐effects model was used to combine the data according to the Mantel‒Haenszel method. Both models provided similar results when interstudy heterogeneity was absent; however, when heterogeneity was high, the random effects model was more appropriate. Heterogeneity across individual studies was assessed via the *I* ^2^ statistic, with *I* ^2^ > 50% considered statistically significant. Publication bias was not formally tested because the number of included studies was small. Subgroup analyses were performed where feasible. Publication bias was assessed via funnel plots of the primary outcome.

## RESULTS

**Figure 1:**
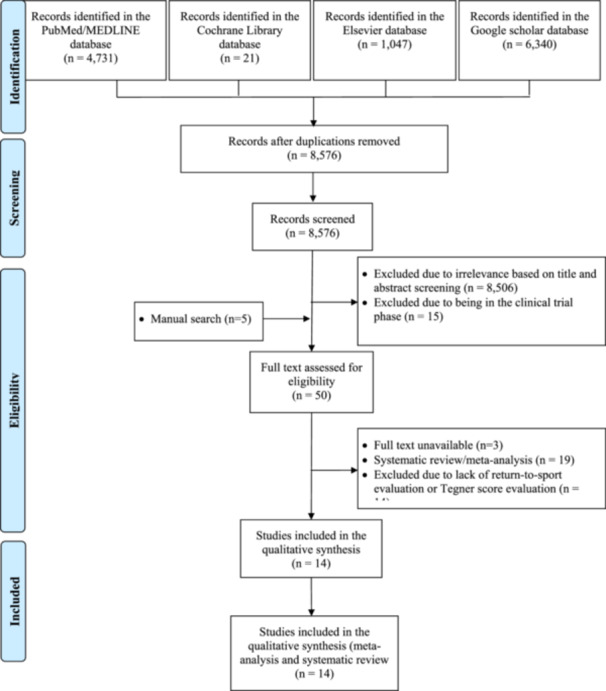
PRISMA (Preferred Reporting Items for Systematic Reviews and Meta‐Analyses) flowchart of the identification and selection of the studies included in the meta‐analysis.

The initial search yielded 12,139 articles. After duplications, titles, abstracts and ongoing clinical trials were excluded, 50 articles remained. This number was reduced to 14 articles after excluding systematic reviews/meta‐analyses and studies for which the full text could not be retrieved. After a full‐text review and manual search, 14 articles (2357 patients) met the eligibility criteria: four studies were randomized controlled trials (RCTs) (549 patients), and 10 were nonrandomized studies (1808 patients). The literature search process is summarized in Figure 1.

**Figure 2:**
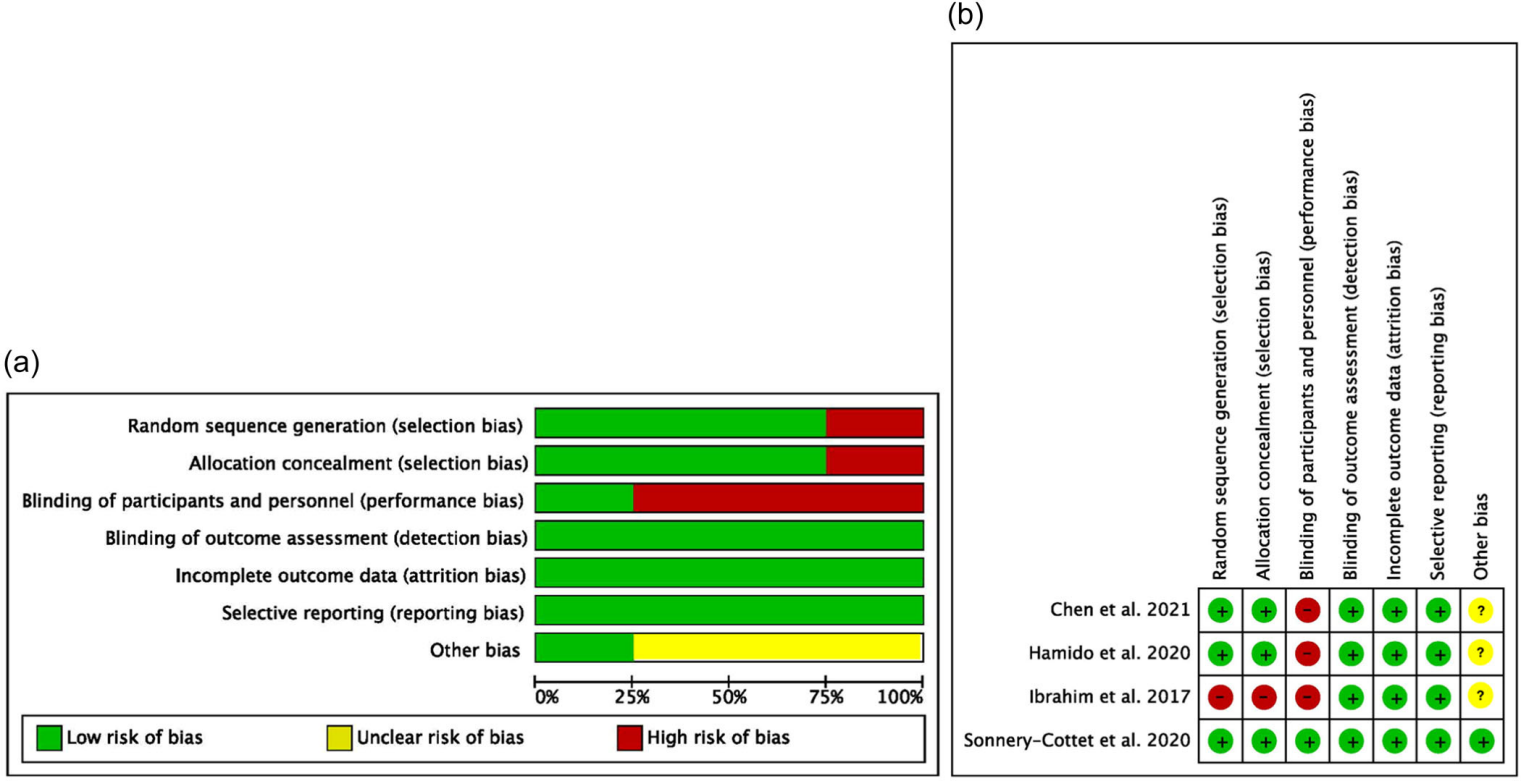
Quality assessment of the included studies. (a) Graph of the risk of different types of bias. (b) Summary of the risk of bias. += low risk of bias; −= high risk of bias; ?= unclear or unknown risk of bias.

**Figure 3:**
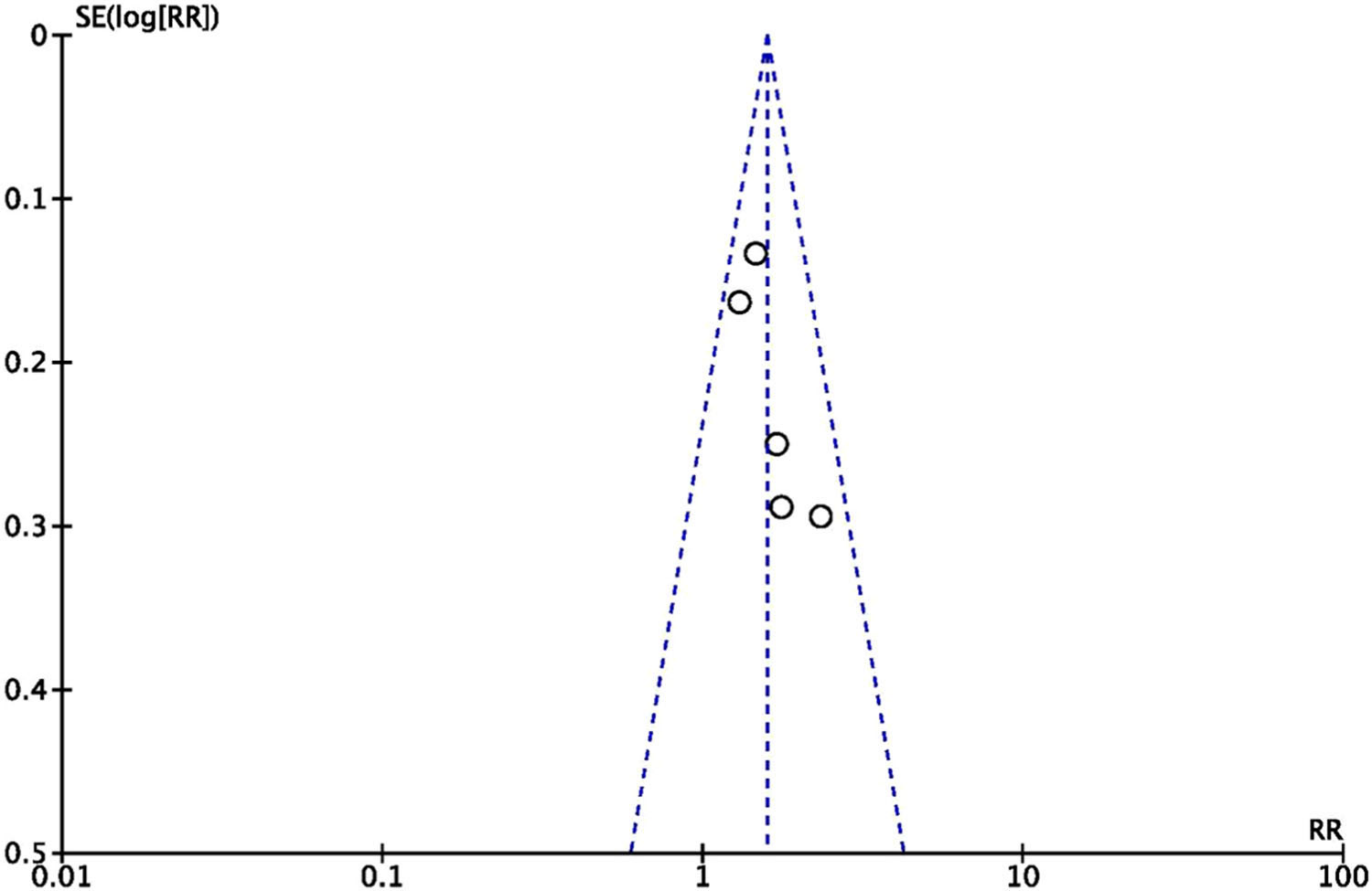
Funnel graph showing the standard error and risk ratios for return to sports.

All the articles were assessed for methodological quality via the Methodological Index for Nonrandomized Studies (MINORS) tool for nonrandomized studies (Table 1). For the RCTs, we used the Cochrane risk‐of‐bias tool (Table 2), with the quality assessment shown in Figure 2a and risk of bias assessment (Figure 2b) and the funnel plot shown in Figure 3. From the total 14 studies, 10 studies were assessed via the MINORS tool and four studies were assessed via the Cochrane risk‐of‐bias tool. Studies with a MINORS score ≤ 12 were excluded because of a high risk of bias. The final sample included 14 studies (Table 3). All the studies adequately reported a succinct study aim and appropriate outcome measures. Any disagreements in the initial ratings of methodological quality between the two reviewers were resolved through discussion until a consensus was reached.

**Table 1:** Quality scoring of the included studies based on the MINORS criteria.

References	MINORS points												Total
	1	2	3	4	5	6	7	8	9	10	11	12	
Sonnery‐Cottet et al. [43]	2	2	2	2	0	2	0	2	2	2	2	2	20
Sonnery‐Cottet et al. [42]	2	2	2	2	2	2	1	2	2	2	2	2	23
Lee et al. [29]	2	2	2	2	2	0	2	2	2	0	2	2	20
Sonnery‐Cottet et al. [40]	2	2	2	2	0	2	2	2	2	2	2	2	22
Zhang et al. [54]	1	1	2	2	0	2	0	1	2	2	2	2	17
Coquard et al. [11]	2	1	2	2	0	0	2	2	2	2	2	2	19
Laboudie et al. [27]	2	2	2	2	0	2	2	2	2	2	2	2	22
Ye et. al. [52]	2	2	2	2	0	2	2	2	2	0	2	2	20
Goncharov et al. [18]	2	0	2	2	0	2	2	0	2	2	0	0	14
Yoon et al. [53]	2	2	2	2	2	2	2	2	2	2	2	2	24

Abbreviation: MINORS, methodological items for nonrandomized studies.

**Table 2:** Cochrane risk‐of‐bias tool for RCTs.

Cochrane risk	Sonnery‐Cottet et al. [41]	Ibrahim et al. [24]	Chen et al. [9]	Hamido et al. [20]
Random sequence generation	Low risk	High risk	Low risk	Low risk
Allocation concealment	Low risk	High risk	Low risk	Low risk
Selective reporting	Low risk	Low risk	Low risk	Low risk
Other bias	Low risk	Unclear risk	Unclear risk	Unclear risk
Blinding of participants and personnel	Low risk	High risk	High risk	High risk
Blinding of outcome assessment	Low risk	Low risk	Low risk	Low risk
Incomplete outcome data	Low risk	Low risk	Low risk	Low risk
**Conclusion**	**High quality**	**Poor quality**	**Fair quality**	**Fair quality**

Abbreviation: RCT, randomized controlled trial.

**Table 3:** Summary of the studies.

References/LOE	Study type	Participants			Primary/revision	ACL technique		ALLR technique			Follow‐up (months)	Fixation angle
		All	ACLR	ACLR + ALLR		Technique	Graft	Femoral insertion	Tibial insertion	Graft		
Zhang et al. [54]/III	Prospective	40	20	20	Primary	SB	ST‐G	Anterior to the LFE	Middle of Gerdy's tubercle and fibular head	ITB	12	30° flexion
	Prospective	40	20	20	Primary	DB	ST‐G	Anterior to the LFE	Middle of Gerdy's tubercle and fibular head	ITB	12	30° flexion
Sonnery‐Cottet et al. [43]/II	Prospective	326	105	221	Primary	SB	BTB	Proximal anterior part of LFE	1. Posterior to the Gerdy's tubercle; 2. anterior to the fibular head	GT	35.4	20° flexion
	Prospective	397	176	221	Primary	SB	ST‐G	Proximal anterior part of LFE	1. Posterior to Gerdy's tubercle; 2. Anterior to the fibular head	GT	35.4	20° flexion
Ibrahim et al. [24]/I	RCT	103	50	53	Primary	SB	ST‐G	Proximal anterior part of LFE	Middle of Gerdy's tubercle and fibular head	GT	27	30° flexion
Sonnery‐Cottet et al. [42]/III	Retrospective	383	194	189	Primary	SB	BTB; ST‐G	Proximal posterior to the LFE	1. Posterior to Gerdy's tubercle; 2. Anterior to the fibular head	GT	12	20° flexion
Lee et al. [29]/III	Retrospective	87	45	42	Revision	SB	Allograft tibialis anterior	Proximal posterior to the LFE	Middle of Gerdy's tubercle and fibular head	GT	38.2	30° flexion
Goncharov et al. [18]/II	Prospective	48	30	18	Primary	SB	BTB	LFE	Middle of Gerdy's tubercle and fibular head	ST/GT	24	90° flexion
Yoon et al. [53]/III	Retrospective	39	21	18	Revision	SB	NR	Proximal posterior to the LFE	Middle of Gerdy's tubercle and fibular head	Allograft tibialis tendon	24	30° flexion
Hamido et al. [20]/I	RCT	102	52	50	Primary	SB	ST‐G	Proximal posterior to the LFE	Middle of Gerdy's tubercle and fibular head	GT	60	0‐15° flexion
Sonnery‐Cottet et al. [41]/I	RCT	224	112	112	Primary	SB	BTB	Proximal posterior to the LFE	1. Posterior to Gerdy's tubercle; 2. Anterior to the fibular head	GT	12	20° flexion
Chen et al. [9]/I	RCT	120	57	63	Primary	DB	ST‐G	Distal posterior to the LFE	Medial to Gerdy's tubercle	GT‐anterior half peroneus longus	24	Full extension
Sonnery‐Cottet et al. [40]/III	Retrospective	172	86	86	Primary	SB	BTB; ST‐G	Proximal posterior to the LFE	1. Posterior to Gerdy's tubercle; 2. Anterior to the fibular head	GT	103	Full extension
Laboudie et al. [27]/III	Retrospective	203	101	102	Primary	SB	ST‐G	Proximal posterior to the LFE	Posterior to Gerdy's tubercle	GT	57	Full extension
Coquard et al. [11]/III	Retrospective	222	111	111	Primary	SB	ST‐G	Proximal posterior to the LFE	1. Posterior to Gerdy's tubercle; 2. Anterior to fibular head	GT	6	Full extension
Ye et. al. [52]/III	Retrospective	92	44	48	Primary	DB	ST‐G	Distal posterior to the LFE	Medial to Gerdy's tubercle	GT‐anterior half peroneus longus	24	Full extension

Abbreviations: ACLR, anterior cruciate ligament reconstruction; ALLR, anterolateral ligament reconstruction; BTB, bone–patellar tendon–bone; DB, double bundle; ITB, iliotibial band; LFE, lateral femoral epycondyle; NR, not reported; RCT, randomized controlled trial; SB, single bundle; ST‐G, semitendinosus‐gracilis.

### Demographics

Among the 2357 patients, 1133 underwent ACLR combined with anterolateral reconstruction and 1224 patients underwent ACLR only (Table 3).

### Return to sports

**Figure 4:**
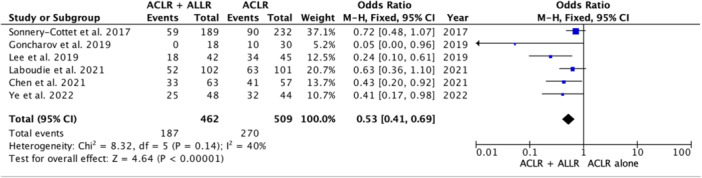
Forest plot of the incidence of failure to return to sports after ACLR. The forest plot shows a significantly lower incidence of failure to return to sport after additional ALLR combined with ACLR than after isolated ACLR. ACLR, anterior cruciate ligament reconstruction; ALLR, anterolateral ligament reconstruction.

Data from 509 patients who underwent ACLR alone and 462 patients who underwent ACLR combined with ALLR were extracted from six studies [9, 18, 27, 29, 43, 52]. Overall, the results revealed that the incidence of patients who were able to return to sports after ACLR combined with ALLR was greater than the incidence after ACLR alone (OR 1.88 [95% CI, 1.44–2.46]; *p* < 0.00001), with a heterogeneity of 0% (Figure 4).

**Figure 5:**
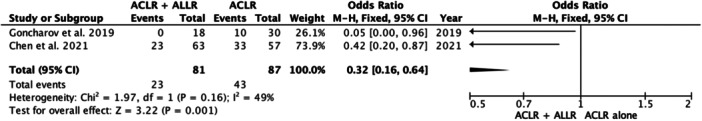
Forest plot of the incidence of failure to return to competition after ACLR. The forest plot shows a significantly lower incidence of failure to return to sport after additional ALLR combined with ACLR than after isolated ACLR. ACLR, anterior cruciate ligament reconstruction; ALLR, anterolateral ligament reconstruction.

**Figure 6:**
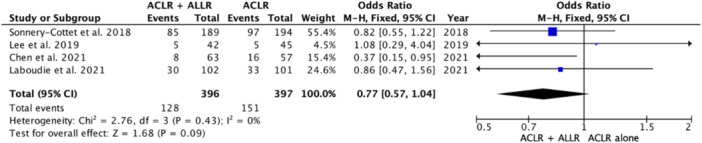
Forest plot of the incidence of failure to return to any sport after ACLR. The forest plot shows that the rate of failure to return to any sport was not significantly different between ACLR with additional ALLR and isolated ACLR. ACLR, anterior cruciate ligament reconstruction; ALLR, anterolateral ligament reconstruction.

We also evaluated the incidence of returning to competition. Among 14 studies, only two evaluated the incidence of return to competition [9, 18]. A comparison of 87 patients who underwent isolated ACLR with 81 patients who underwent ACLR combined with ALLR revealed that the incidence of patients who returned to competition was greater in the combined ACLR and ALLR group than in the isolated ACLR group (OR 3.10 [95% CI, 1.56–6.16]; *p* = 0.001), with a heterogeneity of 0% (Figure 5). The number of patients who were able to return to any type or level of sport did not differ significantly between the ACL combined with ALLR group and the isolated ACLR group (OR 1.24 [95% CI, 0.91–1.67]; *p* = 0.17), *I* ^2 ^= 0%) (Figure 6).

### Patient‐reported outcome measurements

**Figure 7:**
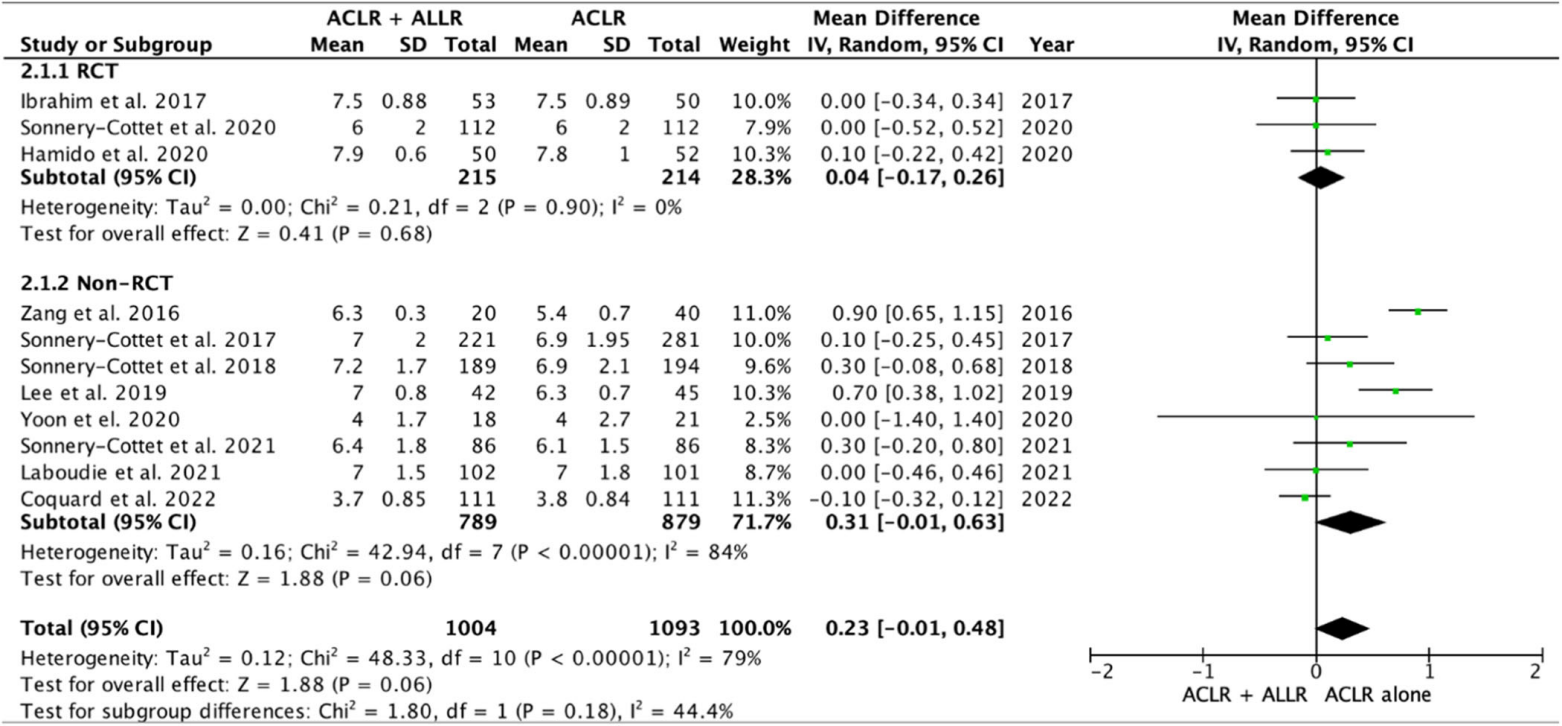
Forest plot comparing the Tegner score after single‐bundle hamstring ACLR. The forest plot shows no significant difference in the Tegner score after single‐bundle hamstring ACLR with additional ALLR versus isolated ACLR. ACLR, anterior cruciate ligament reconstruction; ALLR, anterolateral ligament reconstruction.

**Figure 8:**
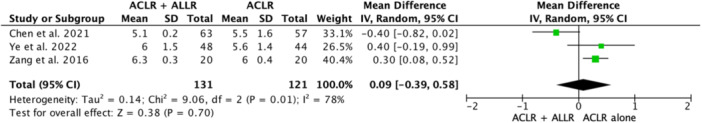
Forest plot comparing the Tegner score after double‐bundle ACLR. The forest plot shows no significant difference in the Tegner score after double‐bundle hamstring ACLR with additional ALLR versus isolated ACLR. ACLR, anterior cruciate ligament reconstruction; ALLR, anterolateral ligament reconstruction.

**Figure 9:**
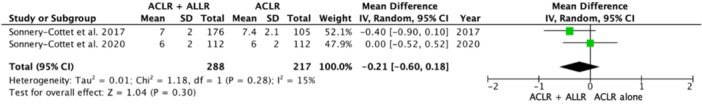
Forest plot comparing the Tegner score after BTB ACLR. The forest plot shows no significant difference in the Tegner score after BTB ACLR with additional ALLR versus isolated ACLR. ACLR, anterior cruciate ligament reconstruction; ALLR, anterolateral ligament reconstruction; BTB, bone–patellar tendon–bone.

**Figure 10:**
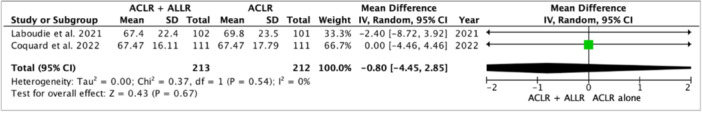
Forest plot comparing the ACL‐RSI scores after ACLR. The forest plot shows no significant difference in the ACL‐RSI after ACLR with additional ALLR versus isolated ACLR. ACLR, anterior cruciate ligament reconstruction; ALLR, anterolateral ligament reconstruction; ACL‐RSI, anterior cruciate ligament‐return to sports injury.

The Tegner scores of 121 patients who underwent isolated ACLR did not differ from those of 131 patients who underwent ACL combined with ALLR, either in terms of the use of a single bundle (Figure 7), double‐bundle hamstring tendon (Figure 8) or bone–patellar tendon–bone (BTB) as the ACL graft (Figure 9). The ACL‐RSI scores of 212 patients after isolated ACLR also did not differ from those of 213 patients who underwent combined ACLR and ALLR (Figure 10).

## DISCUSSION

The most important finding of this study was that more patients who underwent ACL combined with ALLR were able to return to the same level of sports as that prior to injury than patients who underwent ACLR alone. ACL combined with ALLR also resulted in a greater number of patients returning to competition. However, the Tegner score yielded inconsistent findings, especially when comparing combined ACLR and ALLR with isolated single‐bundle ACLR.

The return to preinjury sports levels and participation in competitions have been emphasized as the goals of ACLR [4, 30]. Greater rates of return to sports and competitions were found in the ACLR combined with ALLR group than in the isolated ACLR group. In the present study, a greater proportion of individuals (59.5%) resumed participation in sports after undergoing ACLR combined with ALLR. In contrast, the isolated ACLR group presented a lower rate of return to sports, with only 46.9% of the individuals achieving this outcome. A previous study revealed that the rate of return to sports after ACLR combined with ALLR was greater than that after isolated hamstring ACLR. Nevertheless, no difference was found between isolated BTB ACLR and ACLR combined with ALLR [12]. Interestingly, surgeons increasingly choose to perform ACLR combined with any peripheral reconstruction, either LET or ALL, with an increase from 22.1% to 58.6%, whereas others (12.9%) choose to perform it as a routine procedure [8].

Another important goal of ACLR is to prevent re‐rupture. A 15‐fold increased risk of a second ACL injury has been identified in adolescent athletes who experienced ACL injury and subsequently returned to sports [36]. A previous study by Lai showed that adding the ALL can reduce anterior laxity and graft failure compared with isolated ACLR [28]. Several risk factors are associated with an increased incidence of ACL re‐rupture, including a return to competitive side‐stepping, pivoting or jumping sports and the contact mechanism of the index injury [39]. Other related factors are posterior tibial slope ≥ 10°, KT‐1000 ≥ 3.0 mm, thigh atrophy ≥ 2.50 cm and return to sports < 9.5 months after primary ACLR [19], as well as residual muscle weakness, imbalance and asymmetrical movement and loading patterns [36]. Therefore, residual pivot shift must be prevented. Some surgeons prefer to perform additional anterolateral structure augmentation before performing high‐grade pivot shift and knee hyperextension [45]. The KT‐1000 of patients who had undergone isolated ACLR was lower than that of patients who had undergone ACLR combined with ALLR [21], although no difference was found between patients who had undergone isolated ACLR and those who had undergone ACLR combined with ALLR who had a KT‐1000 ≥ 3.0 mm [48].

LET is another technique to prevent rotational instability. LET and ACL + ALL have comparable clinical outcomes. However, the respective re‐rupture rates in ACLR + ALLR and ACLR + LET were 1.14%, and 4.03% (*p* = 0.015). Therefore, ACLR + LET is associated with a greater risk of re‐rupture [1]. LET has been shown to increase the likelihood of a longer professional career in pivoting sports, with a high rate of RTS [6, 32]. However, the rate of RTS has not been compared between ACL + LET and ACL + ALL. Nevertheless, any AEAP, either LET or ALL, results in a high level of return to play/sport [23].

Psychological factors may also affect the rates of return to sports [34]. Psychological readiness had a moderate to large effect on return to sports. Fear of reinjury is the main reason for changing or refusing return to sports [2, 3, 4]. Psychological readiness is used to describe mental factors that affect return to sports after ACL injury. The patient should aim for a return to sports to allow clinicians can assess psychological readiness and provide proper interventions to reach the patient's return to sports goals. Therefore, the ACL‐RSI was developed to identify patients who struggle with returning to sports [49]. In this study, no difference was found in the ACL‐RSI between isolated ACLR and ACLR combined with ALLR. The addition of ALLR did not increase the psychological readiness of patients.

This study also yielded interesting results. The Tegner score was similar between the ACLR alone and ACLR combined with ALLR groups. Similar results were also reported by Sorensen et al. [41] Although a previous study showed that the Tegner level can predict successful return to sport in ACLR [24], Barret et al. reported that the Tegner activity level is not adequately designed to investigate return to sport [4]. The same results were observed in our study, with no differences in the Tegner score or ACL‐RSI. This is probably due to the psychological readiness and volume training that affect the rate of return to sport, not only anatomical factors [31].

This study had several limitations. First, a limited number of studies with different designs were evaluated. The heterogeneity of the studies, lack of multicentre studies, type of study (RCT or non‐RCT), patient age, preinjury sports level, ACL graft selection, ALL graft selection, ALL technique (attachment points and fixation angle) and postoperative rehabilitation influenced the results of this study. The lack of randomization in non‐RCTs may also be responsible for the heterogeneity. Second, preoperative rotational instability was uncertain in this study. In addition, the pivot‐shift test used to evaluate rotational instability is subjective and has a wide variation in testing manoeuvres. [30] However, it is the most common test for evaluating rotational instability.

## CONCLUSION

Patients who underwent ACLR in combination with ALLR had higher rates of return to sports and competition than patients who underwent ACLR alone. However, the Tegner activity score and ACL‐RSI were comparable between these two groups. This finding may assist surgeons in making decisions when treating patients undergoing ACLR, especially athletes.

## AUTHOR CONTRIBUTIONS

All the authors presently listed have made substantive intellectual contributions to this manuscript through either data collection and analysis, and manuscript production and/or editing. Nicolaas C. Budhiparama and Imelda Lumban‐Gaol have been heavily involved in the data review and manuscript revision process. Nicolaas C. Budhiparama and Imelda Lumban‐Gaol were heavily involved in the study design and data acquisition. Nicolaas C. Budhiparama, Dwikora Novembri Utomo, Imelda Lumban‐Gaol and Dananjaya Putramega involved in manuscript production. Dwikora Novembri Utomo and Krisna Yuarno Phatama were involved in the revision process.

## CONFLICT OF INTEREST STATEMENT

One of the authors, N. C. B., receives payment or benefits from DePuy Johnson & Johnson and Zimmer Biomet and sits on the editorial board of BJJ, CORR, OJSM, *Journal of Orthopaedic Surgery*. The remaining authors declare no conflicts of interest.

## ETHICS STATEMENT

Not applicable.

## Supporting information

Supporting Information.

## Data Availability

The data that support the findings of this study are available from the corresponding author upon reasonable request.
